# MicroRNAs miR-451a and Let-7i-5p Profiles in Circulating Exosomes Vary among Individuals with Different Sickle Hemoglobin Genotypes and Malaria

**DOI:** 10.3390/jcm11030500

**Published:** 2022-01-19

**Authors:** Keri Oxendine Harp, Alaijah Bashi, Felix Botchway, Yvonne Dei-Adomakoh, Shareen A. Iqbal, Michael D. Wilson, Andrew A. Adjei, Jonathan K. Stiles, Adel Driss

**Affiliations:** 1 Department of Physiology, Morehouse School of Medicine, Atlanta, GA 30310, USA; kerioxendine99@yahoo.com (K.O.H.); abashi@msm.edu (A.B.); shareen.iqbal@gmail.com (S.A.I.); 2 Department of Pathology, Korle-Bu Teaching Hospital, University of Ghana Medical School, Accra 4236, Ghana; felixbotchway@gmail.com (F.B.); andrewanthonyadjei@yahoo.com (A.A.A.); 3 Department of Haematology, Korle-Bu Teaching Hospital, University of Ghana Medical School, Accra 4236, Ghana; deiadom@yahoo.com; 4 Department of Parasitology, Noguchi Memorial Institute for Medical Research, University of Ghana, Accra 4236, Ghana; MWilson@noguchi.ug.edu.gh; 5 Department of Microbiology, Biochemistry and Immunology, Morehouse School of Medicine, Atlanta, GA 30310, USA; jstiles@msm.edu

**Keywords:** malaria, hemoglobin genotypes, miRNAs, exosome, hemoglobinopathies, microvesicles, small extracellular vesicles, sEVs, global health, hematology, malaria protection, heme, polymorphisms, liposomes, biomarkers

## Abstract

Sickle cell disease (SCD) occurs when two alleles of mutated hemoglobin (HbS or HbC) are inherited (HbSS and HbSC) rather than one (HbAS or HbAC), which indicates a person carries the sickle cell trait. The high prevalence of these two alleles in Africa have been associated with reduced malaria susceptibility. Recent in vitro research has been shown that microRNAs (miRNAs) miR-451a and let-7i-5p are differentially expressed in HbSS erythrocytes compared to healthy controls (HbAA) and are overexpressed in *Plasmodium*-infected malaria erythrocytes. However, these miRNAs have not been fully examined in the plasma of people with different sickle hemoglobin genotypes. Plasma circulating miRNAs are commonly encapsulated in extracellular vesicles, such as exosomes, and are thought to play a role in disease development. Circulating exosomal miR-451a and let-7i-5p were quantified from individuals with various hemoglobin genotypes (HbAA, HbAS, HbAC, HbSS, HbSC, and HbCC) with (+) and without (−) malaria. The results showed a higher level of exosomal let-7i-5p and miR-451a in HbSS-. Exosomal let-7i-5p and miR-451a levels were lower in HbSS+ compared to other genotypes. Based on the area under the curve (AUC) of the Receiver Operating Characteristics (ROCs), both exosomal miRNAs may be useful disease biomarkers for SCD with malaria. Finally, miR-451a and let-7i-5p modulate genes involved in inflammation, making them potential biomarkers of pathogenesis for both diseases.

## 1. Introduction

Sickle cell disease (SCD) is one of the most common severe monogenic disorders in the world associated with clinical complications, such as stroke, infections, acute splenic sequestration, acute chest syndrome, pulmonary hypertension, and leg ulcers [1]. SCD is a genetic disorder associated with a mutation in the beta-chain in both hemoglobin (Hb) alleles that cause the red blood cell (RBC) morphology to change under hypoxic conditions [1]. The Hb allele variant S (HbS) causes sickle cell anemia (SCA) [2,3]. HbS and HbC are associated with SCA when both HbS and/or HbC alleles are inherited (HbSS or HbSC). While sickle cell trait (SCT) occurs when one HbS or HbC allele is inherited from one parent and one normal allele (HbA) from another parent (HbAS or HbAC). People with SCT often have no complications from carrying the HbS or HbC allele [4]. The host hemoglobin genotype has been shown to play a protective role against malaria [5,6]. However, the mechanism by which sickle hemoglobin genotypes provide protection is poorly understood. In 2018, there were 405,000 malaria deaths worldwide, with most occurring among children [7]. SCD and malaria require treatment strategies that target pathophysiological mechanisms and clinical complications. Functional biomarkers must be identified and evaluated for their diagnostic and prognostic value.

Studies have shown that HbSS erythrocytes have differential expression of many erythrocytic microRNAs (miRNAs) [8,9]. While there are hundreds of miRNAs, two miRNAs have been identified as being associated with HbSS and HbAS, as well as parasite growth in vitro [3,9]. Researchers have discovered elevated levels of miR-451a and let-7i-5p in HbSS and HbAS erythrocytes, which inhibit parasite growth in vitro [8,9]. MiRNAs are small, non-coding RNAs of approximately 20 to 25 nucleotides long that endogenously regulate gene expression post-transcriptionally [10]. MiRNAs are produced by various cells, and a single miRNA can have multiple targets [11]. The integrity of circulating miRNAs is maintained in plasma when encapsulated in microvesicles such as exosomes (vesicles 30–100 nm in size) [12]. Exosomes contain biomolecules such as miRNA, mRNA, proteins, and lipids. Exosomes transfer these biomolecules from one cell to another [13]. Exosomes and their contents have been used to develop new diagnostics, drugs, and vaccines [12,14]. Exosomal miRNAs have been used as biomarkers in cancer and chronic and infectious diseases [12,15,16]. There are hundreds of highly regulated miRNAs in the body that bind to target mRNAs and control translation [17]. While miRNAs have been shown to be differentially expressed in SCD patients compared to non-SCD controls, their exosomal miRNAs have not been characterized [9]. Likewise, miRNAs have been examined for malaria, but much research is still needed to fully understand the role of exosomal miRNAs in malaria pathogenesis [18].

Based on previous in vitro studies, we chose to evaluate exosomal miR-451a and let-7i-5p in our cohort [3,9]. While other studies have investigated endogenous miRNA levels in cell culture, our study specifically focused on exosomal miRNA levels. Studying exosomal miRNAs, we can learn more about how miRNAs are involved in SCD and malaria pathogenesis, and their findings may be used to develop therapeutic approaches. Therefore, in this study, we evaluated the expression of exosomal miR-451a and let-7i-5p among persons with or without malaria, with one (HbAS and HbAC), two (HbSS, HbSC, and HbCC), or no copies (HbAA) of the Hb allele variant S or C. We hypothesized that miR-451a and let-7i-5p are differentially expressed in plasma exosomes isolated from individuals with different hemoglobin variants and may contribute to the pathogenesis of both SCD and malaria. Studying miRNA expression levels in exosomes, specifically, miR-451a and let-7i-5p, and their relationship to inflammation in SCD will enhance our understanding of SCD and malaria pathogenesis. In addition, analyzing the effect of malaria and SCD on exosomal miRNA could prompt the development of severity biomarkers.

## 2. Materials and Methods

### 2.1. Study Population

The data were collected from the Greater Accra region of Ghana, West Africa, at the Korle-Bu Teaching Hospital and district hospitals, namely, Princess Marie Louise Children’s Hospital, Korle-Bu Polyclinic, Mamprobi Polyclinic, Ussher Polyclinic, Shai-Osudoku, and LA General Hospital. Volunteers were recruited between February and November 2014 and from June 2017 to July 2019 as part of an ongoing National Institute of Health’s Fogarty International Center-funded SCD and a malaria study between Morehouse School of Medicine and the University of Ghana.

Ethical approval was obtained from the University of Ghana’s Noguchi Memorial Institute for Medical Research and College of Health Sciences, and the Morehouse School of Medicine (Atlanta, GA) institutional review boards before the study’s commencement. Participants of age ≥ 18 years gave their informed consent. Among persons aged < 18 years, parents or guardians provided written consent. Exclusion criteria included individuals with abnormal fetal Hb (HbF), pregnant women, insufficient complete blood count (CBC) information, or positive HIV test. All samples were numerically coded to anonymize the data. A total of 183 subjects were randomly selected from a pool of a total 923 volunteers. Of the 183 subjects, the average age of all subjects was close to 30 years, representing all 6 sickle hemoglobin genotypes (HbAA, HbAS, HbAC, HbSS, HbSC, and HbCC) with (+) and without (−) malaria. Several groups were hard to recruit, so we used all individuals recruited for those groups, regardless of age and gender. There were ten individuals for the HbCC−, HbAS+, and HbAC+ groups and eleven individuals for the HbAC− and HbSS+ groups. For the other genotype groups, there were 35 HbAA−, 31 HbAS−, 17 HbSS−, 15 HbSC−, 26 HbAA+, and 7 HbSC+. During the entire recruitment period, we were unable to identify any individuals with a HbCC+ genotype, so that group could not be included in the study.

### 2.2. Laboratory Evaluation of Blood Samples

Blood samples were collected using BD Vacutainer^®^ CPT™ (BD Bioscience, San Jose, CA, USA) Mononuclear Cell Preparation Tubes. Genotyping patients for sickle cell status was conducted at the Department of Haematology at the Korle-Bu Teaching Hospital using cellulose acetate membrane electrophoresis using HbAA, HbSS, and HbCC controls to determine participants’ hemoglobin status [19]. The medical questionnaires used for enrollment were consistent in all facilities where patients’ clinical and laboratory data were obtained. CBC counts were obtained by the hospital or clinical pathology laboratories from venous blood samples to determine patient hematological characteristics. Plasma, white blood cells (WBCs), and red blood cells (RBCs) were separated within hours of the collection, as instructed by the CPT tube manufacturer’s protocol [19,20]. Blood samples were tested for *Plasmodium falciparum* using a Rapid Diagnostic Test (RDT) kit and thick smear microscopy. The malaria RDT kits used (First Response^®^ Malaria Ag. pLDH/HRP2 Combo Card Test, WHO reference number: PQDx 0285-010-00) detected both *Plasmodium falciparum*-specific protein HRP2, as well as Pan (Pan lactate dehydrogenase (LDH)), which detects multiple malaria species. HIV status was determined with RDTs (First Response^®^ HIV-1–2 kits). We refer to malaria-infected individuals with (+) and non-infected individuals with (−) after the Hb genotypes.

### 2.3. Exosomal RNA Extraction

Exosomes were isolated from plasma using the Total Exosome Isolation kit (from plasma) (Invitrogen) following the manufacturer’s instructions, and total RNA was isolated from exosomes using the Total Exosome RNA/Protein Isolation kit (Invitrogen) following the manufacturer’s instructions. Samples were stored at −80 °C until further analysis.

### 2.4. Real-Time Quantitative PCR (RT-qPCR)

RNA was reverse transcribed using TaqMan MicroRNA Assay (Applied Biosystems, Waltham, MA, USA) and the TaqMan MicroRNA Reverse Transcription Kit (Applied Biosystems). RT-qPCR was performed using the Roche LightCycler 480 (Roche Applied Science) and the Bio-Rad CFX96 Real-Time PCR System using TaqMan MicroRNA Assay and TaqMan Universal Master Mix II, no UNG (Applied Biosystems) [21]. We focused on miR-451a and let-7i-5p due to the role of their expression role in SCD and malaria [3,9]. The RT-qPCR primers for miR-451a, let-7i-5p, and U6 (used as an internal control) were designed using Invitrogen MicroRNA Analysis and the TaqMan Assay search tool. U6 snRNA has been widely used in other studies as an internal control for exosomal miRNA [22,23]. The ΔΔCT method was used to evaluate the relative fold change in miRNA expression in Excel (Microsoft Office 365) and normalized to U6 expression [24]. ΔΔCt was created using RNU6 as an endogenous control and HbAA- as the control group in Excel using the Livak method [24]. ΔΔCt values were log-transformed to normalize the data. Any ΔΔCt over three standard deviations from the mean were flagged and removed as outliers.

### 2.5. Statistical Analysis

All statistical analyses were performed in GraphPad PRISM version 7.04 for Windows (GraphPad Software). A sample size calculation was performed using a 95% confidence interval and power of 80, using preliminary RT-qPCR miRNA data, which determined that a minimum of 6 samples per group was needed. Descriptive statistics were used to illustrate the study samples and hematologic profiles, stratified by Hb subtypes. Normality was determined through the D’Agostino and Pearson method. When comparing more than two groups, ANOVA and Tukey’s multiple comparison tests were used to assess within- and between-group’s significance among variables. However, to compare exosomal miRNAs levels between individual genotypes with and without malaria, an unpaired t-test was used. MiR-451a and let-7i-5p logged ΔΔCTs were correlated using Pearson correlation for each genotype and malaria status, as well as independent of genotype. MiR-451a and let-7i-5p ΔΔCTs were correlated with blood characteristics using Pearson correlation by genotype. A *p*-value of *p* < 0.05 was considered significant for all tests.

### 2.6. Receiver Operating Characteristic (ROC) Curve Analysis

To investigate the potential use of miRNAs as diagnostic tools, the ROC was calculated. Area under the curve (AUC) was calculated to show the accuracy of each analysis with *p*-values. Analyses were completed using GraphPad Prism software (GraphPad 9.2.0). Statistical significance was established at *p* < 0.05 for all tests.

### 2.7. Bioinformatics

To identify and evaluate potential gene targets of miR-451a and let-7i-5p, each miRNA was assessed with five different statistical algorithms that enlist predicted targets (DIANA-TarBase v7.0 [25], RNA22 [26], mirDB [27,28], TargetScan [29], microRNA.org [25,26,27,28,29,30]). All five lists generated were converted into a Venn diagram using software (http://bioinformatics.psb.ugent.be/webtools/Venn/, last accessed 23 May 2021). The software determined targets that overlapped across the prediction algorithms used. The predicted targets’ gene ontology was analyzed with Integrated Pathways Analysis (IPA) and FunRich tool (Version 3.1.3) (https://www.FunRich.org, last accessed 23 May 2021) [31,32].

## 3. Results

**Figure 1:**
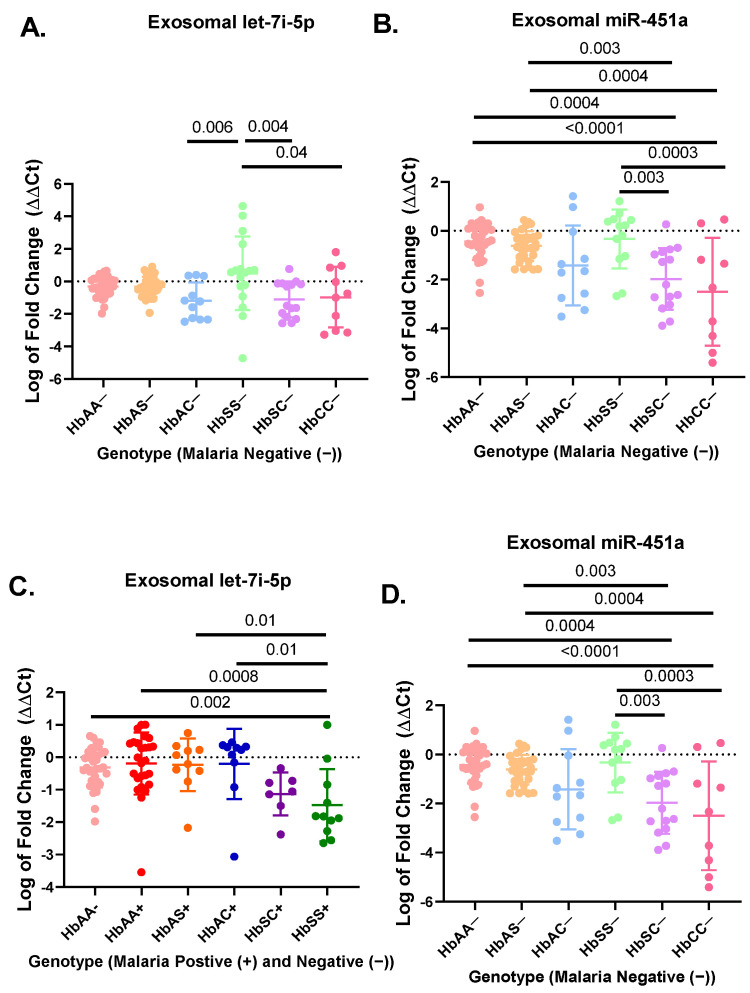
RT-qPCR results for exosomal miR-451a and let-7i-5p expressed as log of fold change (ΔΔCt) in a bar graph expressing mean and standard deviation. ΔΔCt was created using RNU6 as endogenous control and HbAA− as the control group in Excel using the Livak method [28] (**A**) There was a significant difference in relative expression of exosomal let-7i-5p for HbSS− vs. HbAC− (*p* = 0.006), HbSS− vs. HbCC− (*p* = 0.04), and HbSS− vs. HbSC− (*p* = 0.004). (**B**) There was a significant difference for expression of exosomal miR-451a between HbAA− vs. HbCC− (*p* < 0.0001), HbAA− vs. HbSC− (*p* = 0.0004), HbAS− vs. HbCC− (*p* = 0.0004), HbAS− vs. HbSC− (*p* = 0.003), HbCC− vs. HbSS− (*p* = 0.0003), and HbSC− vs. HbSS− (*p* = 0.003). (**C**) There was a significant difference for exosomal let-7i-5p between HbAA− vs. HbSS+ (*p* = 0.002), HbAA+ vs. HbSS+ (*p* = 0.0008), HbAC+ vs. HbSS+ (*p* = 0.01), and HbAS+ vs. HbSS+ (*p* = 0.01). (**D**) There was a significant difference for exosomal miR-451a between HbSS+ vs. HbAA− (*p* < 0.0001), HbSS+ vs. HbAA+ (*p* < 0.0001), HbSS+ vs. HbAS+ (*p* < 0.0001), HbSC+ vs. HbSS+ (*p* = 0.04), and HbSS+ vs. HbAC+ (*p* < 0.0001).

A total of 183 participant samples were randomly selected out of a cohort of 923 participants from each genotype (HbAA, HbAS, HbAC, HbSC, HbSS, and HbCC) with (+) and without (−) malaria (Table 1). The hematological characteristics and clinical data of the original cohort of 923 participants were compared in previous publication [33]. Results from the miRNA RT-qPCR were analyzed by ΔΔCt analysis. For the non-malaria groups, exosomal let-7i-5p was significantly upregulated in HbSS− patients as compared to HbAC− (*p* = 0.006), HbCC− (*p* = 0.04), and HbSC− (*p* = 0.004) (Figure 1A). Exosomal miR-451a levels were significantly upregulated in HbSS- compared to HbSC− (*p* = 0.003) and HbCC− (*p* = 0.0003) (Figure 1B). Additionally, exosomal miR-451a was significantly reduced for HbSC− compared to HbAA− (*p* = 0.0004) and HbAS− (*p* = 0.003). Finally, exosomal miR-451a was significantly reduced for HbCC− compared to HbAA− (*p* < 0.0001) and HbAS− (*p* = 0.0004) (Figure 1B). In the malaria positive cohorts, exosomal miR-451a levels were significantly reduced in HbSS+ compared to HbAA− (*p* < 0.0001), HbAA+ (*p* < 0.0001), HbAS+ (*p* < 0.0001), HbAC+ (*p* < 0.0001), and HbSC+ (*p* = 0.04) (Figure 1D). While in malaria-positive groups, exosomal let-7i-5p was significantly reduced in HbSS+ compared to HbAA− (*p* = 0.002), HbAA+ (*p* = 0.0008), HbAS+ (*p* = 0.01), and HbAC+ (*p* = 0.01) (Figure 1C).

There was no difference in exosomal let-7i-5p and miR-451a levels between HbAA, HbAS, HbAC, and HbSC groups with and without malaria, except for HbSS−, which was elevated compared to HbSS+ (*p* < 0.02) (Figure S1G,H). Overall, this suggests that exosomal miR-451a and let-7i-5p may play a role in SCD and malaria inflammation.

### 3.1. Let-7i-5p and miR-451a Expression Correlate with Each Other Regardless of Genotype and Malaria Status

**Figure 2:**
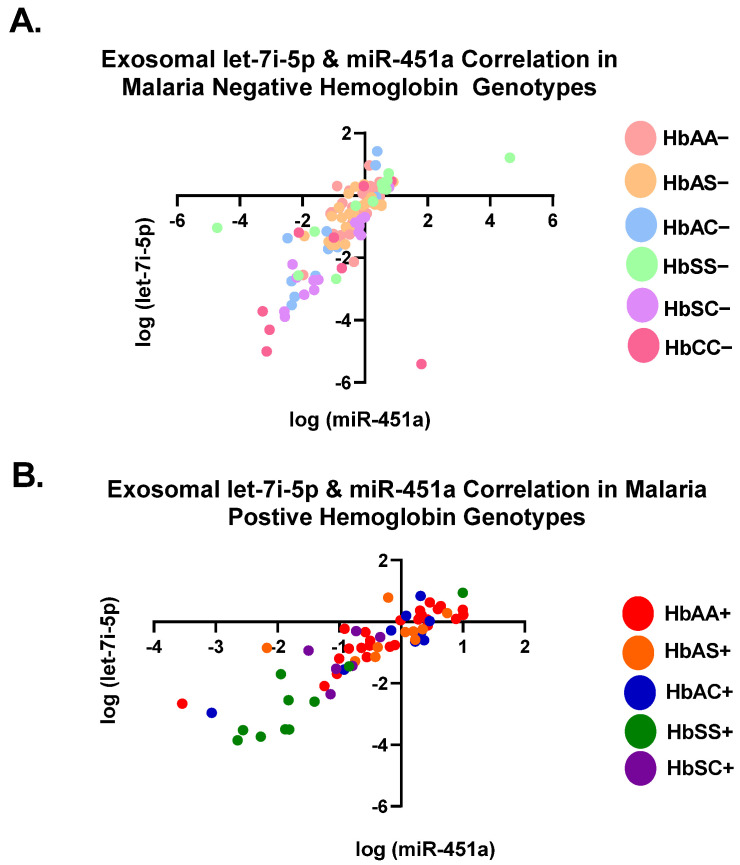
Correlation of exosomal let-7i-5p and miR-451a expression levels among all genotypes. Two-tailed Pearson correlation with 95% confidence bands of let-7i-5p and miR-451a between genotypes. (**A**) For HbAA−, HbAS−, HbSS−, HbAC−, HbSC−, and HbCC−, R^2^ = 0.45 and *p* < 0.0001. (**B**) For HbAA+, HbAS+, HbSS+, HbAC+, and HbSC+, R2 = 0.76 and *p* < 0.0001.

Pearson correlation analysis showed that the expression levels of both exosomal let-7i-5p and miR-451a were correlated with each genotype independently (Table 2). All genotypes with and without malaria were significantly (*p* < 0.05) correlated between the two miRNAs, except for HbAS+, HbSC+, and HbCC− (Table 2). When all Hb groups were combined, there was a significant correlation with R^2^ = 0.45 and *p* < 0.0001 for malaria-negative groups and R^2^ = 76 and *p* < 0.0001 for malaria-positive groups. (Table 2, Figure 2). Additionally, when all genotypes, regardless of malaria status, were combined, there was a significant correlation between the two miRNAs (R^2^ = 0.54, *p* < 0.0001) (Table 2). For all genotypes with and without malaria, except HbAS+, HbSC+, and HbCC−, miR-451a and let-7i-5p significantly correlated with each other.

**Figure 3:**
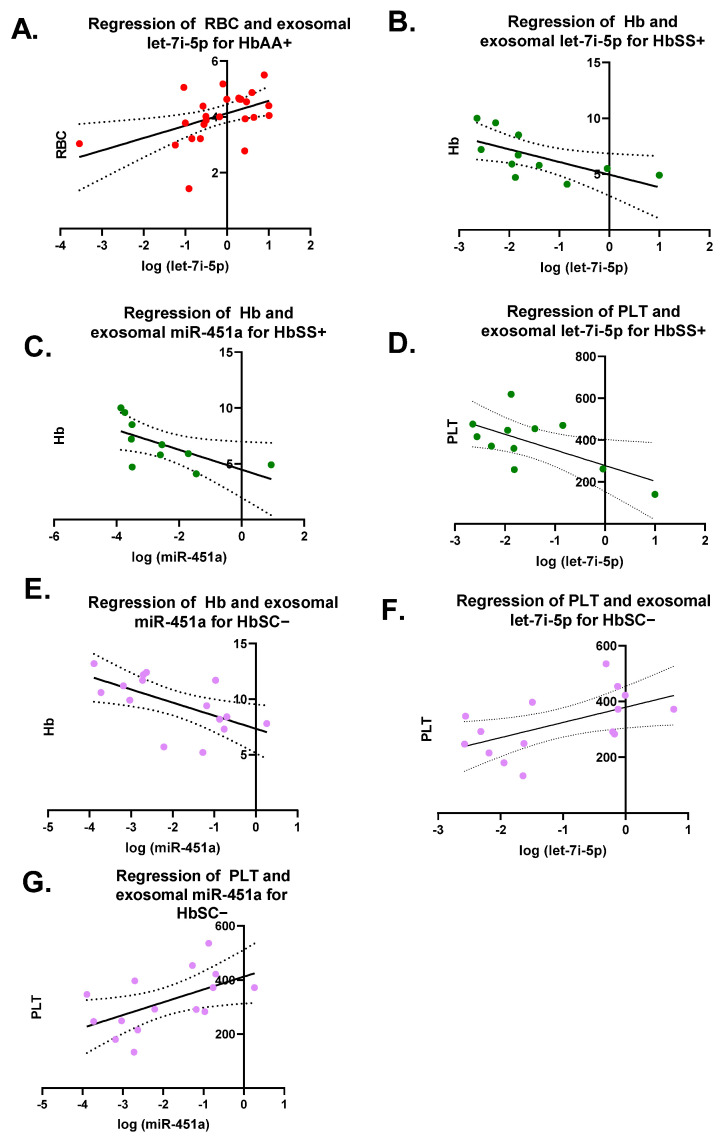
Pearson correlation on each miRNA versus complete blood counts (CBCs) (RBC, WBC, Hb, and PLT) for each genotype with and without malaria. RBCs = red blood cells; WBCs = white blood cells; Hb = hemoglobin; PLT = platelet. Only 7 correlations were significant and shown. (**A**) For HbAA+, correlation between exosomal let-7i-5p and RBC resulted in R^2^ = 0.23 and *p* = 0.01. (**B**) For HbSS+, correlation between exosomal let-7i-5p and Hb was R^2^ = 0.4 and *p* = 0.04. (**C**) For HbSS+, correlation between exosomal miR-451a and Hb was R^2^ = 0.42 and *p* = 0.04. (**D**) For HbSS+, correlation between exosomal let-7i-5p and PLT was R^2^ = 0.4 and *p* = 0.04. (**E**) For HbSC−, correlation between exosomal miR-451a and Hb resulted in R^2^ = 0.36 and *p* = 0.02. (**F**) For HbSC−, correlation between exosomal let-7i-5p and PLT resulted in R^2^ = 0.31 and *p* = 0.03. (**G**) For HbSC−, correlation between exosomal miR-451a and PLT resulted in R^2^ = 0.3 and *p* = 0.03.

Pearson correlation analyses were performed on each miRNA versus CBC values (RBC, WBC, Hb, and PLT) for each genotype with and without malaria (Figure 3). There were seven significant correlations (Figure 3). For HbAA+, there was a significant correlation between exosomal let-7i-5p and RBCs levels with R^2^ = 0.23 and *p* = 0.01 (Figure 3A). There was a significant correlation for HbSS+ between exosomal let-7i-5p and Hb levels, as well as PLT counts with a R^2^ = 0.4 and *p* = 0.04 and R^2^ = 0.4 and *p* = 0.04, respectively (Figure 3B–D). Additionally, for HbSS+, there was a significant correlation between exosomal miR-451a and Hb levels with R^2^ = 0.42 and *p* = 0.04. HbSC− also had a significant correlation between exosomal miR-451a and Hb with R^2^ = 0.36 and *p* = 0.02 (Figure 3E). Similarly, HbSC− showed a significantly correlation between exosomal let-7i-5p and miR-451a and PLTs with R^2^ = 0.31 and *p* = 0.03 and R^2^ = 0.3 and *p* = 0.03, respectively (Figure 3F,G).

### 3.2. miR-451a and Let-7i-5p Are Significant Biomarkers for Sickle Cell Anemia and Malaria Status

**Figure 4:**
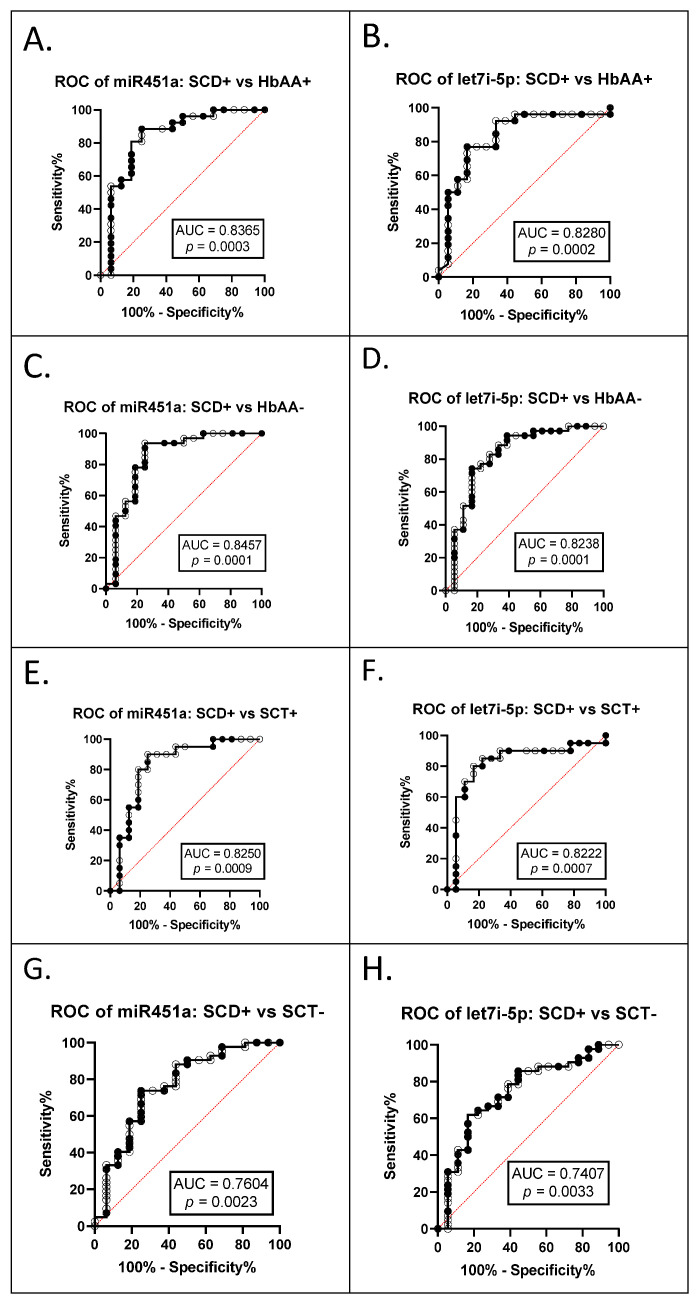
ROC analysis for exosomal miR-451a and let-7i-5p (**A**) miR-451a, SCA+ (HbSS+ and HbSC+ combined) vs. HbAA+, AUC = 0.8365, *p* = 0.0003; (**B**) let-7i-5p, SCA+ vs. HbAA+, AUC = 0.8280, *p* = 0.0002; (**C**) miR-451a, SCA+ vs. HbAA−, AUC = 0.8457, *p* = 0.0001; (**D**) let-7i-5p, SCA+ vs. HbAA−, AUC = 0.8238, *p* = 0.0001; (**E**) miR-451a, SCA+ vs. SCT+ (HbAS+ and HbAC+ combined), AUC = 0.8250, *p* = 0.0009; (**F**) let-7i-5p, SCA+ vs. SCT+, AUC = 0.8222, *p* = 0.0007; (**G**) miR-451a, SCA+ vs. SCT− (HbAS− and HbAC− combined), AUC = 0.7604, *p* = 0.0023; (**H**) let-7i-5p, SCA+ vs. SCT−, AUC = 0.7407, *p* = 0.0033.

The AUC of the ROCs were calculated to investigate whether exosomal miR-451a and let-7i-5p can be used as potential diagnostic markers (Table 3). The ROC curves and AUC estimates enabled us to predict that both exosomal miRNAs can distinguish between SCA+ (HbSS+ and HbSC+ combined) and HbAA+ (miR-451a AUC 0.8365, *p* = 0.0003; let-7i-5p AUC 0.828, *p* = 0.0002) (Figure 4A,B); between SCA+ and HbAA− (miR-451a AUC 0.8457, *p* = 0.0001, let-7i-5p AUC 0.8238, *p* = 0.0001) (Figure 4C,D); between SCA+ and SCT+ (HbAS+ and HbAC+ combined) (miR-451a AUC 0.8250, *p* = 0.0009, let-7i-5p AUC 0.8222, *p* = 0.0007) (Figure 4E,F); and between SCA+ and SCT− (HbAS− and HbAC− combined) (miR-451a AUC 0.7604, *p* = 0.0023, let-7i-5p AUC 0.7407, *p* = 0.0033) (Figure 4G,H).

### 3.3. Predictions of Let-7i-5p and miR-451a Gene Targets

MiRNAs are known to have multiple targets, and multiple algorithms predict targets for miRNAs. To determine if there were any predicted targets of interest for let-7i-5p and miR-451a for further investigation, we used multiple prediction algorithms. Therefore, potential gene targets of let-7i-5p and miR-451a were determined using five commonly used prediction algorithms (DIANA, mirDB, RNA22, TargetScan, and microRNA.org). The predicted targets were organized in Venn diagrams (Figure S2) to show the distribution of target genes. Lin-28 Homolog B (*LIN28B*) and Fidgetin (*FIGN*) were predicted as let-7i-5p’s target genes by all five algorithms. None of the five algorithms predicted common targets for miR-451a. Using Ingenuity Pathway Analysis (IPA), twelve out of all the predicted targets for let-7i-5p had connections with the Let-7 miRNAs family or each other. These genes are Adrenoceptor Beta 2 (*ADRB2*), Hypoxia Inducible Factor 1 Subunit Alpha Inhibitor (HIF1AN), RAN Binding Protein 2 (*RANBP2*), E2F Transcription Factor 5 (*E2F5*), NRAS Proto-Oncogene, GTPase (*NRAS*), Lysine methyltransferase 2D (*KMT2D*), Fibronectin Type III Domain Containing 3A (FNDC3A), High Mobility Group AT-Hook 2 (*HMGA2*), MSM4, P53 Regulator (*MDM4*), Tripartite motif Containing 71 (*TRIM71*), Lin-28 Homolog B (*LIN28B*), and Insulin-Like Growth Factor Binding Protein 1 (*IGFBP1*) (Figure S3). All predicted targets of miR-451a in IPA are directly connected to miR-451a. These genes are Odd-Skipped Related Transcription Factor 1 *(OSR1*), Sterile Alpha Motif Domain-Containing 4B (*SAMD4B*), and Protease Subunit Beta 8 (*PSMB8*) (Figure S3). All the predicted targets identified in IPA have direct or indirect relationships with inflammation signaling pathways via *STAT3* and *NFҡB* (Figure S3). These results suggest miR-451a and let-7i-5p targets of interest may alter pathogenesis through inflammatory pathways. Molecular function, cellular components, and biological processes were analyzed in FunRich to look for any similarity between predicted targets (Figure S3). Three different molecular functions were predicted for miR-451a: transcription factor activity (33%), ubiquitin-specific protease activity (33%), and molecular function unknown (33%). Let-7i-5p predicted targets represent four distinct molecular functions: transcription factor activity (~18%), transcription regulator activity (~8%), ubiquitin-specific protease activity (~8%), and unknown molecular function (~16%) (Figure S3). The predicted gene targets were further analyzed according to cellular components. MiR-451a predicted targets include five cellular components: nucleolus (50%), cytoplasm (50%), exosomes (50%), proteasome complex (50%), and nucleus (100%). The predicted targets for let-7i-5p represent four cellular components: nucleolus (~21%), cytoplasm (~55%), exosomes (3%), and nucleus (~66%). Finally, the predicted targets were stratified for biological processes. The predicted targets of miR-451a represent three biological processes: regulation of nucleobase, nucleoside, nucleotide, and nucleic acid metabolism (~33%), protein metabolism (~33%), and unknown biological process (~33%). Finally, let-7i-5p predicted targets represent five biological processes: signal transduction (~18%); cell communication (~18%); regulation of nucleobase, nucleoside, nucleotide, and nucleic acid metabolism (~34%); protein metabolism (~10%); and biological process unknown (~10%) (Figure S4). These results suggest that the predicted targets have a variable range of functions and may alter pathways other than inflammation in SCD pathogenesis. More studies are needed to investigate miR-451a and let-7i-5p targets’ effects on inflammation in malaria and SCD.

## 4. Discussion

Our study identified associations between Hb genotype and the expression of exosomal miRNA. These miRNAs could mediate the severity of malaria and sickle cell disease, which could indicate how SCT might protect against malaria infection. We hypothesized that miR-451a and let-7i-5p are differentially expressed in plasma exosomes isolated from individuals with different hemoglobin variants and may contribute to the pathogenesis of both SCD and malaria. We found that exosomal let-7i-5p was significantly upregulated in HbSS− patients compared to HbCC−, HbAC−, and HbSC−, while exosomal miR-451a was also significantly elevated in HbSS− compared to HbSC− and HbCC− (Figure 1). However, for exosomal miR-451a, HbSC− and HbCC− were significantly decreased compared to other genotypes without malaria. However, for exosomal miR-451a and let-7i-5p, we observed a shift from significant elevation without malaria to a considerable decrease in HbSS+ compared to other genotypes. Finally, our gene target analysis indicated that endogenous let-7i-5p and miR-451a are involved in inflammation associated with SCD severity. Our results suggest that these miRNAs may play an essential role in regulating the pathogenesis of SCD and malaria.

When measuring exosomal let-7i-5p and miR-451a expression levels, we found a statistically significant (*p* < 0.05) increased expression in HbSS− patients compared to other genotypes (Figure 1). Let-7i-5p expression levels were similar to those measured in a previous study, which measured non-exosomal miRNA expression in RBC culture and found that miRNA expression was associated with parasite growth [3]. There has also been an association reported between let-7i-5p and malaria in vitro and in the plasma of children in Mozambique [34]. Although the study in Mozambique did not examine exosomal miRNA and only compared uncomplicated to complicated malaria, it did not determine what the levels of miRNAs would be in non-malaria participants [34]. Additionally, the study in Mozambique did not investigate the sickle cell status, and we know miRNA levels are altered by sickle cells status from other studies [3,9]. In our cohort, we also found an inverse relationship between RBC count and measured exosomal let-7i-5p levels in HbAA+ in our cohort (Figure 3). Furthermore, the AUC of the ROC curves of exosomal miR-451a and let-7i-5p showed the potential to be used as biomarkers to discriminate SCA, SCT, and HbAA when complicated by malaria (Figure 4). However, the sensitivity and specificity were not remarkably high. In this study, there were relatively few patient samples, so further analysis of these exosomal miRNAs needs to be conducted.

Other miRNAs, such as miR-144 and miR-451a, have been reported to be associated with RBC characteristics and hemolysis [8,35]. We also found correlations between Hb levels and miRNA levels in both HbSS+ (let-7i-5p and miR-451a) and HbSC− (miR-451a) groups (Figure 3). HbSS is associated with reduced RBC survival as hemolysis is increased due to sickling [36]. Additionally, RBC lifespans are reduced in individuals with HbSS due to other factors such as oxidative stress, which cause membrane damage, increased cell rigidity, and cell dehydration, resulting in increased hemolysis [36].

To investigate the relationship between clinical characteristics and exosomal miRNA expression, we examined possible targets of let-7i-5p and miR-451a. Five different prediction algorithms and IPA enabled us to identify three potential common targets (*OSR1*, *SAMD4B*, and *PSMB8*) for miR-451a and 12 predicted targets for let-7i-5p. Out of the three predicted miR-451a common targets, only PSMB8 has been reported to be associated with miR-451a and involved in inflammation [37]. *PSMB8*, also known as *LMP7*, inhibits NFҡB when levels of miR-451a are high [37].

Using IPA, twelve predicted target genes of let-7i-5p, *ADRB2, HIF1AN, RANBP2, E2F5, NRAS, KMT2D, FNDC3A, HMGA2, MDM4, TRIM71, LIN28B,* and *IGFBP1*, were connected, directly or indirectly, to the let-7 miRNAs family and other targets (Table 4). Out of the twelve targets, five (*ADRB2, HIF1AN, HMGA2, TRIM71,* and *LIN28B)* were associated with SCD pathogenesis (Table 4). *ADRB2* has been reported to be linked to the adhesion of RBCs in HbSS [38,39,40]. In *ADRB2*, polymorphisms can increase the adhesion of RBCs in HbSS, which can cause vaso-occlusive crisis and stroke [38,39,40]. *HIF1AN* is considered a master regulator in hypoxia pathways, and painful vaso-occlusive crisis is thought to be caused by multiple occurrences of hypoxia in SCD patients [41,42]. A SCD patient became transfusion independent after treatment with globin lentiviral gene therapy, which caused a chromosomal integration of the vector at the *HMGA2* site [43]. *TRIM71* is an orthologue of *LIN-41*, which is a highly conserved target for all the let-7 family miRNAs [44]. *LIN28B* regulates fetal hemoglobin (HbF) via the let-7 family [45]. Increasing HbF levels is a potential SCD therapy. Hydroxyurea, a current medication for SCD, increases HbF levels [46]. These results suggest a more comprehensive look into *PSMB8* as potential targets for miR-451a and *ADRB2, HIF1AN, HMGA2, TRIM71,* and *LIN28B* as potential targets for let-7i-5p.

Further analysis in FunRich to assess the molecular function, cellular components, and biological process classified the majority of the predicted targets of let-7i-5p and miR-451a into four molecular functions: transcription regulator activity, transcription factor activity, ubiquitin-specific protease activity, and unknown molecular function. The majority of the predicted targets were associated with five cellular components: nucleolus, cytoplasm, exosomes, proteasome complex, and nucleus. Further analysis revealed that most of the predicted targets were involved in five biological processes: signal transduction, cell communication, regulation of nucleobase, nucleoside, nucleotide, and nucleic acid metabolism, protein metabolism, and unknown biological process. Let-7i-5p and miR-451a play roles in multiple biological pathways. Thus, they could modulate SCD and malaria pathogenesis via multiple pathways, such as the inflammatory pathway, especially in HbSS and HbSC.

Moreover, miR-451a and let-7i-5p have been shown to be highly enriched in HbAS and HbSS erythrocytes in vitro [3]. MiR-451 and let-7 family of miRNAs have been shown to control key regulators of genes involved in immune function [57,58,59]. MiR-451a enhances erythroid differentiation and is involved in oxidative stress. Let-7i-5p acts directly on the 3′-UTR of BTB Domain and CNC Homolog 1 (BACH1), associated with heme binding, and negatively regulates the expression of this protein and thereby upregulates heme oxygenase-decycling 1 (HMOX1) gene expression [57,60,61]. HMOX1 gene codes for the Heme-oxygenase-1 (HO-1), the rate-limiting enzyme in the degradation of heme groups to biliverdin, carbon monoxide (CO), and iron. Increased free heme produced during malaria infection induces inflammation, which damages the host vascular endothelium and exacerbates fatal malaria pathogenesis [62,63,64,65]. As a cellular stress regulator, HO-1 protects against oxidative stress, heavy metal toxicity, UV radiation, inflammation, and prevents the deleterious effects of heme, as well as mediates anti-inflammatory and anti-apoptotic functions [66,67]. HO-1 induced by reactive oxygen species and nitric oxide (NO) is involved in regulating angiogenesis [68,69]. HO-1 facilitates the repair of injured tissues through the inhibition of infiltrating inflammatory cells [70]. In SCA, the HbS genotype causes hemolytic anemia, leading to the accumulation of high levels of cell-free Hb and free heme in plasma [71,72]. HbAS individuals (sickle cell trait) also accumulate low (non-pathologic) levels of heme in plasma [73]. Free heme in the erythrocytes has been implicated in damage of the red cell membrane, leading to higher red cell adhesion to the endothelium in SCA [74]. Higher frequency and severity of sickle cell crises is related to enhanced release of free heme, exhibited as membrane-bound iron [75]. Free heme plays a crucial role in SCA’s pathophysiology as a cofactor of HbS polymerization [76]. Once released from Hb, a phenomenon favored in HbS [77], free heme becomes cytotoxic [78,79,80]. In SCA patients, miR-451a and let-7i-5p overexpression could be behind the chronic inflammation observed as well as the harmful consequences of high levels of free heme due to abnormal HO-1 expression.

The samples used for this study were selected from a larger cohort, which compared hematological parameters between HbAA, HbAS, HbAC, HbSC, HbSS, and HbCC with and without malaria [33]. The study determined that Hb genotypes and malaria status changed an individual’s hematological parameters. For example, SCA individuals, regardless of malaria status, had increased WBC and platelet counts and lower Hb levels compared to other Hb genotypes [33]. Additionally, the same pool of samples was also used previously to measure cytokine and chemokine levels [81]. The study explored the possibility of using inflammatory cytokines as biomarkers between Hb genotypes and malaria [81]. In particular, IL-6, which let-7i-5p is known to target, was able to independently discriminate between HbAA− vs. HbAA+, HbAC− vs. HbAC+, and HbAS+ vs. HbAC+ [81]. We also found that the levels of other inflammatory cytokines were significantly different between genotypes with and without malaria [81]. A cross-examination of cytokine and miRNA levels was not possible due to the small sample size. Based on the significant association between SCD and malaria, these miRNAs may be able to help manage their pathogenesis.

The present study reveals significant changes in exosomal let-7i-5p and miR-451a levels and their association with SCD and malaria, but some limitations need to be considered. First, parasite densities of malaria-positive individuals were not explored, which may lead to systematic bias since other studies have shown parasite densities vary by Hb genotypes [82,83]. The gold standard for determining malaria infection is the RDT and thick blood smear. In this study, those with semi-immunity to malaria infection were not taken into consideration due to the limitations of the thick blood smear and rapid diagnostic tests. To remove this disparity, future experiments will use serological tests, such as an ELISA, to determine malaria antibody concentrations and confirm the presence of malaria in those who show a negative blood smear test but are still infected with the disease. Additionally, the severity of malaria was not categorized by severe or uncomplicated, which may have resulted in statistical bias. We do, however, report WBC counts and Hb levels, which are associated with inflammation and anemia, respectively, and are involved with SCD and malaria pathogenesis and severity. Additionally, this study illustrates a possible association between exosomal miRNA levels and inflammation via identifying potential targets and hematological characteristics. Instead, the article opens the discussion for further investigation into how these miRNAs directly impact inflammation in SCD and malaria. Finally, we only analyzed two of the miRNAs of interest in exosomes found in previous studies identifying RBC endogenous miRNAs. However, other circulating miRNAs have been identified in other studies [3,9,34]. To fully understand the role of exosomal miRNAs in malaria and SCD, further studies are needed to evaluate let-7i-5p and miR-451a, as well as other exosomal miRNAs.

## 5. Conclusions

In summary, exosomal let-7i-5p and miR-451a were differentially expressed in HbSS and HbSC. The predicted targets identified that let-7i-5p and miR-451a are associated with host inflammatory pathways. Our findings suggest that exosomal let-7i-5p and miR-451a may play a role in SCD and malaria pathogenesis. These miRNAs potentially modulate SCD and malaria pathogenesis by targeting inflammatory pathways via *NFҡB*. Understanding the relationship between exosomal miR-451a and let-7i-5p, SCT, SCD−induced-inflammatory responses, and malaria-induced-inflammatory responses may lead to novel approaches to interventions. Future interventions may include artificially encapsulated exosomes or liposomes with miRNAs, which could protect against malaria pathogenies and SCA severity.

## Figures and Tables

**Table 1:** Clinical characteristics of study populations. All genotypes with (+) and without (−) malaria—mean and standard deviation for clinical characteristics. White blood cells (WBCs). Red Blood cells (RBCs).

Characteristics (Mean ± SD)	HbAA− *n* = 35	HbAS− *n* = 31	HbAC− *n* = 11	HbSS− *n* = 17	HbSC− *n* = 15	HbCC− *n* = 10	HbAA+ *n* = 26	HbAS+ *n* = 10	HbAC+ *n* = 10	HbSS+ *n* = 11	HbSC+ *n* = 7
Age (years)	37.9 ± 15	38.2 ± 13.1	31.5 ± 11.3	26.3 ± 9.4	32.9 ± 11	34 ± 9.2	33.1 ± 20.7	22 ± 22.4	22.5 ± 12.8	22.5 ± 10.4	38.4 ± 18.9
Sex (male)	17	7	4	8	6	5	13	3	3	6	3
WBC (×10^3^/mm^3^)	6.4 ± 3.5	5.6 ± 1.7	6 ± 1.7	12.11 ± 4.5	10 ± 4.2	8 ± 1.8	6.2 ± 3.5	5.6 ± 2.4	6.2 ± 2	14.1 ± 4.1	10.2 ± 9.6
RBC (×10^6^/µL)	4.4 ± 0.6	4.2 ± 0.6	4.7 ± 1.2	3.3 ± 0.9	3.7 ± 1.1	4.5 ± 0.5	4 ± 0.2	4.3 ± 0.7	4.3 ± 0.6	2.5 ± 1	3.5 ± 1.5
Hemoglobin (g/dL)	12.1 ± 2.6	11.9 ± 1.5	12.3 ± 1.5	8.1 ± 1.1	9.6 ± 2.5	11.8 ± 1.6	11 ± 2.7	11.3 ± 2.1	11.5 ± 1.3	6.6 ± 2	8.6 ± 3.7
Platelets (×10^3^/µL)	266.3 ± 95.5	238.8 ± 69.3	280.1 ± 121.3	435.8 ± 120.4	319.3 ± 108.8	209.5 ± 63	149 ± 89.7	190.6 ± 115.4	167.2 ± 39.7	388.5 ± 131	279.9 ± 139.4

**Table 2:** Assessment of correlation between miR-451a and let-7i-5p in each genotype. Assessment of R^2^ and *p* values of miR-451a and let-7i-5p expression levels in each genotype with and without malaria using Pearson correlation analysis. All malaria-negative groups include HbAA−, HbAS−, HbAC−, HbSC−, HbSS−, and HbCC−. All malaria-positive groups include HbAA+, HbAS+, HbAC+, HbSS+, HbSC+. All Hb genotypes include: HbAA−/+, HbAS−/+, HbAC−/+, HbSC−/+, HbSS−/+, HbCC−. Bold text denotes significance.

Genotype	R^2^	*p*-Value
**HbAA−***n* = 35	0.44	**<0.0001**
**HbAS−***n* = 31	0.53	**<0.0001**
**HbAC−***n* = 11	0.78	**0.0003**
**HbSS−***n* = 17	0.49	**0.007**
**HbSC−***n* = 15	0.9	**<0.0001**
**HbCC−***n* = 10	0.1	0.4
**HbAA+***n* = 26	0.8	**<0.0001**
**HbAS+***n* = 10	0.24	0.15
**HbAC+***n* = 10	0.8	**0.0005**
**HbSS+***n* = 11	0.86	**0.0001**
**HbSC+***n* = 7	0.21	0.36
**All Malaria Negative***n* = 119	0.45	**<0.0001**
**All Malaria Positive***n* = 64	0.76	**<0.0001**
**All Groups***n* = 183	0.54	**<0.0001**

**Table 3:** Data from ROC Analysis for exosomal miR-451a and let-7i-5p. Area under the curve (AUC) and *p*-values of all phenotypic differences with and without malaria for miR-451a. SCA+ (HbSS+ and HbSC+ combined), SCA− (HbSS− and HbSC− combined), SCT+ (HbAS+ and HbAC+ combined), SCT− (HbAS− and HbAC− combined), HbAA−, and HbAA+. Bold text denotes significant values.

	miR-451a		Let-7i-5p	
Groups	AUC	*p*-Value	AUC	*p*-Value
**SCA− vs. SCA+**	0.6586	0.0832	**0.6927**	**0.0249**
**SCA− vs. HbAA−**	0.6395	0.0640	0.5397	0.6059
**SCA− vs. HbAA+**	0.6490	0.0603	0.5152	0.8310
**SCA− vs. SCT+**	0.6268	0.1376	0.5281	0.7349
**SCA− vs. SCT−**	0.5531	0.4537	0.5595	0.3827
**SCA− vs. HbCC−**	0.6825	0.1034	0.5781	0.3470
**SCA+ vs. HbAA−**	**0.8457**	**0.0001**	**0.8238**	**0.0002**
**SCA+ vs. HbAA+**	**0.8365**	**0.0003**	**0.8280**	**0.0001**
**SCA+ vs. SCT+**	**0.8250**	**0.0009**	**0.8222**	**0.0007**
**SCA+ vs. SCT −**	**0.7604**	**0.0023**	**0.7407**	**0.0033**
**SCA+ vs. HbCC−**	0.5556	0.6506	0.5500	0.5987
**HbAA− vs. HbAA+**	0.5282	0.7133	0.5747	0.3213
**HbAA− vs. SCT+**	0.5414	0.6482	0.5077	0.9294
**HbAA− vs. SCT−**	0.6328	0.0515	0.6241	0.0873
**HbAA− vs. HbCC−**	**0.7639**	**0.0167**	0.6154	0.1837
**HbAA+ vs. SCT+**	0.5529	0.5423	0.6164	0.1539
**HbAA+ vs. SCT−**	0.6351	0.0627	0.5724	0.2759
**HbAA+ vs. HbCC−**	**0.7735**	**0.0157**	0.5971	0.2341
**SCT+ vs. SCT−**	0.5982	0.2141	0.6452	0.0662
**SCT+ vs. HbCC−**	0.7667	0.2370	0.6150	0.2134
**SCT− vs. HbCC−**	**0.7143**	**0.0454**	0.5571	0.4698

**Table 4:** Predicted targets of miR-451a and let-7i-5p using IPA analysis. Target gene name, abbreviation, Entrez gene ID, and short description of function.

miRNA Species	Target Gene Name	Abbreviation	Entrez Gene ID	Function	Ref.
**miR-451a**	Odd-Skipped Related Transcription Factor 1	OSR1	130497	Key component in regulation of intracellular concentration of chloride which is required for cell volume regulation.	[47]
	Sterile Alpha Motif Domain- Containing 4B	SAMD4B	55095	Regulator of transcriptional signaling activity.	[48]
	Protease Subunit Beta 8	PSMB8	5696	Inhibits NFҡB when miR-451a levels are raised. Also known as LMP7.	[37]
**Let-7i-5p**	Adrenoceptor Beta 2	ADRB2	154	Can increase the adhesion of HbSS RBCS	[38,39,40]
	Hypoxia Inducible Factor 1 Subunit Alpha Inhibitor	HIF1AN	55662	Helps maintain cell viability during oxygen deprivation	[41,42]
	RAN Binding Protein 2	RANBP2	5903	Is a nuclear pore protein that is involved in the cell cycle	[49]
	E2F Transcription Factor 5	E2F5	1875	Has an important role in cell cycle and tumor suppression	[50]
	NRAS Proto-Oncogene, GTPase	NRAS	4893	Involved in the RAS signaling pathway	[51]
	Lysine methyltransferase 2D	KMT2D	8085	Involved in multiple functions like differentiation & metabolism	[52]
	Fibronectin Type III Domain Containing 3A	FNDC3A	22862	Encodes for Fibronectin module type III (FN3) which mediates protein-protein interactions	[53]
	High Mobility Group AT-Hook 2	HMGA2	8091	A report of a viral vector integrating with an intragenic site in a SCD patient and the SCD patient became transfusion independent	[43]
	MDM4, P53 Regulator	MDM4	4194	Regulates tumor suppressor p53	[54]
	Tripartite motif Containing 71	TRIM71	131405	Is an ortholog of Lineage Variant 41 (LIN-41). Lin-41regulates fetal hemoglobin (HbF) via let-7 miRNAs	[44]
	Lin-28 Homolog B	LIN28B	389421	Regulates HbF via let-7 miRNAs	[45,46]
	Insulin Like Growth Factor Binding Protein 1	IGFBP1	3484	Associated with metabolism	[55,56]

## Data Availability

For original data, please contact adel.driss@gmail.com.

## Supplementary Materials

The following are available online at https://www.mdpi.com/article/10.3390/jcm11030500/s1, Figure S1: Comparisons of each Hb genotype with and without malaria individually for exosomal let-7i-5p and miR-451a levels, Figure S2: Venn Diagram illustrating predicted overlapping targets of miRNAs (let-7i-5p and miR-451a), Figure S3: Pathway interactions of predicted and known miRNA targets in IPA, Figure S4: Predicted targets, for miR-451a and let-7i-5p analyzed in FunRich.
