# Supplementary material for: MicroRNAs miR-451a and Let-7i-5p Profiles in Circulating Exosomes Vary among Individuals with Different Sickle Hemoglobin Genotypes and Malaria
Source: J Clin Med. 2022 Jan 19;11(3):500. doi: 10.3390/jcm11030500 (PMC8837188; doi:10.3390/jcm11030500)
Supplement: Supplementary file 1 [file jcm-11-00500-s001.zip › Supplemental figures and tables JCM_AD_AD-1.pdf]

Figure S1

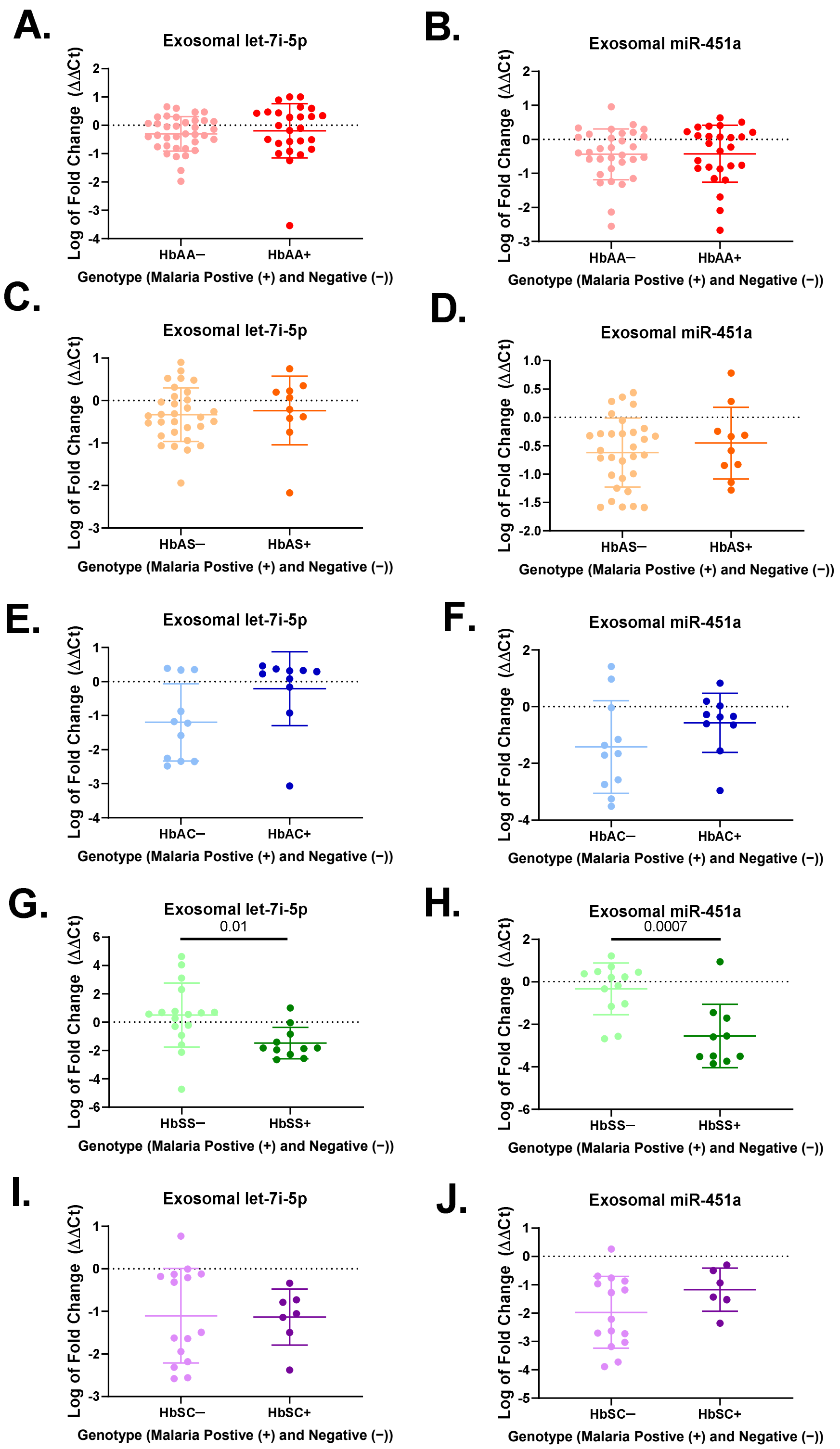

Figure S1: Comparisons of each Hb genotype with and without malaria individually for exosomal let-7i-5p and miR-451a levels. **A.** There was no significant difference in exosomal let-7i-5p between HbAA− vs HbAA+. **B.** There was no significant difference in exosomal miR-451a between HbAA− vs HbAA+. **C.** There was no significant difference in exosomal let-7i-5p between HbAS− vs HbAS+. **D**. There was no significant difference in exosomal miR-451a between HbAS− vs HbAS+. **E.** There was no significant difference in exosomal let-7i-5p between HbAC− vs HbAC+. **F**. There was no significant difference in exosomal miR-451a between HbAC− vs HbAC+. **G.** Exosomal let-7i-5p were elevated in HbSS− compared to HbSS+ (P=0.01). **H**. Exosomal miR-451a levels were elevated in HbSS−

compared to HbSS+ (P=0.0007). **I.** There was no significant difference in exosomal let-7i-5p between HbSC− vs HbSC+. **J**. There was no significant difference in exosomal miR-451a between HbSC− vs HbSC+.

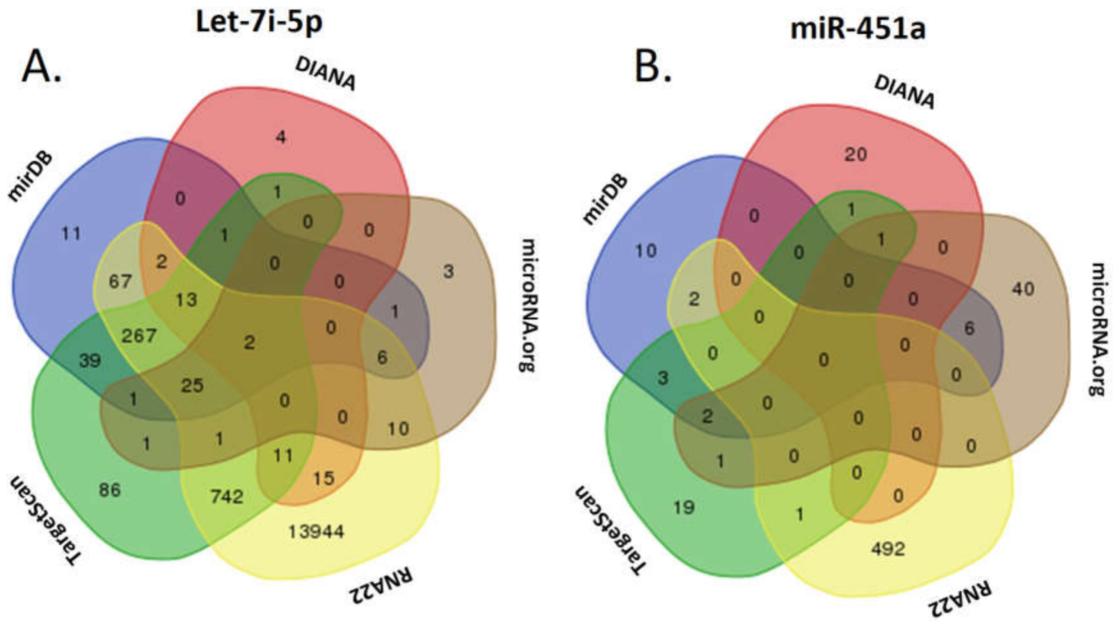

Figure S2: Venn Diagram illustrating predicted overlapping targets of miRNAs (**A.** let-7i-5p and **B.** miR-451a). Five prediction algorithms [DIANA-TarBase v7.0 [25], RNA22 [26], mirDB [27,28], TargetScan [29], microRNA.org [25-30]] were used to identify potential targets for each miRNA.

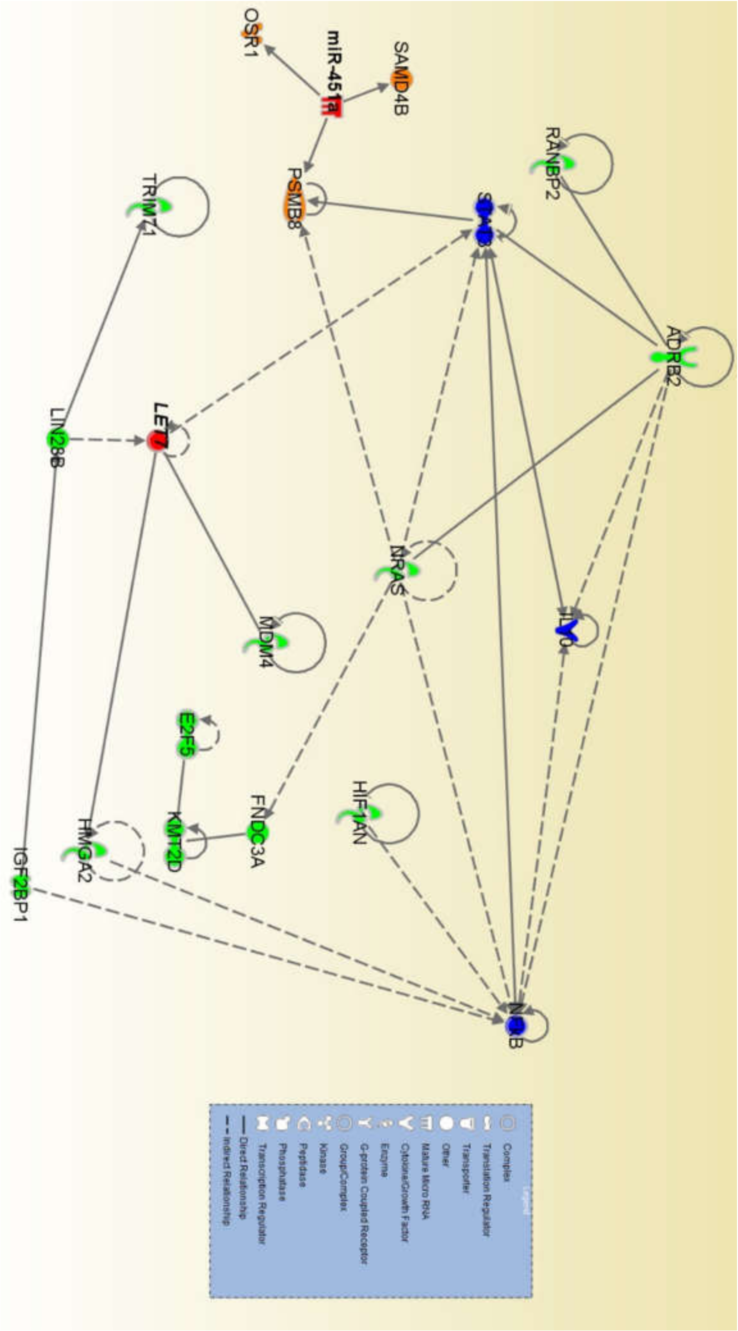

Figure S3: Pathway interactions of predicted and known miRNA targets in IPA. IPA= Ingenuity Pathway Analysis. Only predicated targets with connections are displayed. MiRNA is labeled red, known interaction in other diseases are in blue. Predicted targets for miR-451a are in orange and predicted targets for let-7 are in green.

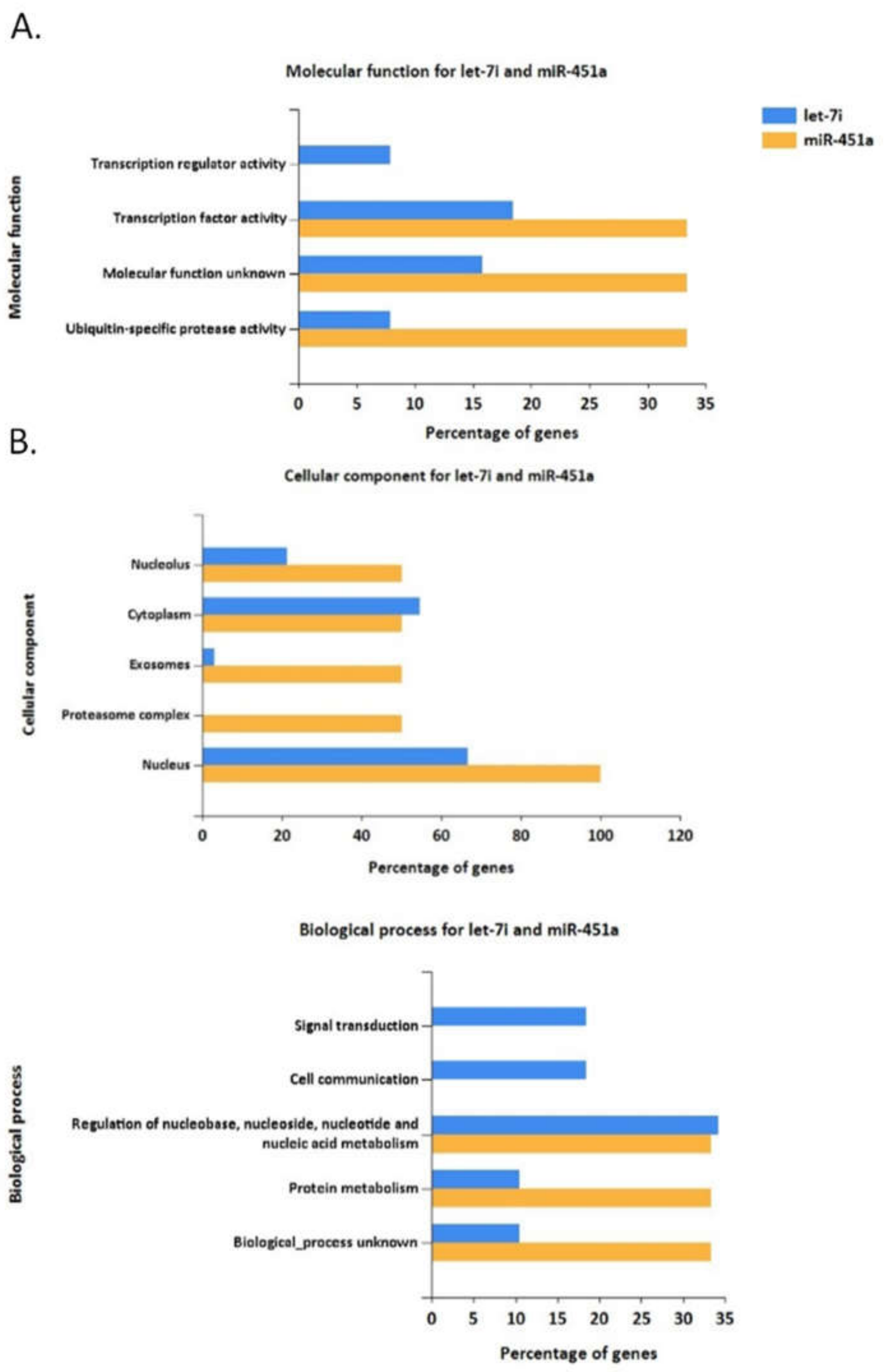

Figure S4: Predicted targets, for miR-451a and let-7i-5p analyzed in FunRich. A. MiR-451a and let-7i-5p predicted targets were stratified according to molecular function. MiR-451a predicted targets represent 3 different molecular functions: transcription factory activity (33%), ubiquitin-specific protease activity (33%), and molecular function unknown (33%). Let-7i-5p predicted targets represent 4 different molecular functions: transcription factory activity (~18%), transcription regulator activity (~8%), ubiquitin-specific protease activity (~8%), and molecular function unknown (~16%). B. MiR-451a and let-7i-5p predicted targets were arranged according to cellular component. MiR-451a predicted targets represent 5 cellular components: nucleolus (50%), cytoplasm (50%), exosomes (50%), proteasome complex (50%), and nucleus (100%). Let-7i-5p predicted targets represented 4 cellular components: nucleolus (~21%), cytoplasm (~55%), exosomes (3%), and nucleus (~66%). C. MiR-451a and let-7i-5p predicted targets were separated according to biological process.

MiR-451a predicted targets represent 3 biological processes: Regulation of nucleobase, nucleoside, nucleotide, and nucleic acid metabolism (~33%), protein metabolism (~33%), and biological process unknown (~33%). Let-7i-5p predicted targets represent 5 biological processes: signal transduction (~18%), cell communication (~18%), regulation of nucleobase, nucleoside, nucleotide, and nucleic acid metabolism (~34%), protein metabolism (~10%), and biological process unknown (~10%).
